# Does the toxicity of endocrine therapy persist into long-term survivorship?: Patient-reported outcome results from a follow-up study beyond a 10-year-survival

**DOI:** 10.1007/s10549-022-06808-9

**Published:** 2022-11-23

**Authors:** Albertini Carmen, Oberguggenberger Anne, Sztankay Monika, Egle Daniel, Giesinger Johannes, Meraner Verena, Hubalek Michael, Brunner Christine

**Affiliations:** 1 Department of Psychiatry, Psychotherapy, Psychosomatics and Medical Psychology, CL Service, University Hospital of Psychiatry II, Medical University of Innsbruck, Innrain 52, A-6020 Innsbruck, Austria; 2 Department of Obstetrics and Gynecology, Medical University of Innsbruck, Anichstrasse 35, A-6020 Innsbruck, Austria

**Keywords:** Breast cancer, Endocrine therapy, Long-term breast cancer survivors, Quality of life, Toxicity, Patient-reported outcomes

## Abstract

**Background:**

Endocrine treatment (ET) is a highly effective breast cancer treatment but can distinctly impair breast cancer patients’ quality of life (QOL). In a patient-reported outcome (PROs) study conducted by the authors in 2011, patients reported higher ET-induced symptom levels than known from the registration trials, and was underestimated. Based on these study results, we investigated the long-term sequelae of ET reported by breast cancer survivors (BCS) in a follow-up study conducted 5–10 years after an earlier assessment.

**Methods:**

BCS who had participated in the earlier study (*n* = 436) were approached for study participation either at one of their routine follow-up appointments or via mail; consenting patients were asked to completed the same PRO assessment used in the original study (FACT-B + ES). BCS with relapse/ progressive disease were excluded from the analysis. We compared long-term endocrine symptomatology and overall QOL outcome (i.e. FACT-G and -ES sum score).

**Results:**

A final sample of 268 BCS was included in the analysis. BCS reported a significant improvement of the overall endocrine symptomatology (baseline mean = 59 vs. follow-up mean = 62, *p* < 0.001), physical (baseline = 23.9 mean vs. follow-up mean = 24.8, *p* < 0.01) and functional well-being (baseline mean = 21.7 vs. follow-up mean = 22.7, *p* = 0.013) and overall QOL (mean baseline = 88.3 vs. mean follow-up = 90.9, *p* = 0.011). However, the prevalence of particular symptoms, well-known to be ET induced, did not change over time such as joint pain (baseline = 45.5% vs. 44.2%, n.s. difference), lack of energy (36.4% vs 33.8%, n.s. difference), weight gain (36.8% vs. 33.9%, n.s. difference) or vaginal dryness (30.2% vs. 33%, n.s. difference) and the proportion reporting lack of interest in sex increased (40.4% vs. 48.7%, *p* < 0.05).

**Conclusion:**

Presented results indicate that BCS recover well in terms of overall endocrine symptomatology and quality of life but experience some clinically relevant and unfavorable ET-related long-term effects.

## Introduction

With constantly increasing survival rates over the last decade, the group of long-term breast cancer survivors (i.e. permanent survivorship according to ASCO, www.cancer.net) has been expanding. Personalized treatments such as endocrine therapy (ET) applied for multiple years after initial treatment make a distinct contribution to these increased survival rates. More than 75% of women diagnosed with breast cancer would receive at least 5 years of ET as part of their treatment [1, 2]. Though increasing survival, women are undergoing these highly effective treatments at the cost of an (potential) enduring impairment of their quality of life (QOL) [3, 4]. Hot flashes, joint pain, sexual problems or emotional instability are among the most prevalent ET side effects challenging patients’ QOL [5, 6]. Some evidence claims these ET treatment side effects and QOL impairments to occur not only during treatment but to persist after treatment completion far into the survivorship stage [7–9]. Hence, survivorship issues such as QOL including not only physical but also psychosocial recovery in the long-term gain importance when it comes to comprehensive survivorship care [10–13]. An essential step in this regard is the systematic identification of ET long-term sequelae most detracting to breast cancer survivors (BCS) including the patient’s subjective experience. For this purpose, patient-reported outcomes (PROs) have been proven to give comprehensive insight into the patient’s physical and psychosocial health complementing provider-generated information [14]. In a study called PRO-BETh (PROs in Breast cancer patients undergoing ET), performed 2009–2011, the authors were able to demonstrate the value of PROs for the understanding of ET treatment toxicity [14, 15]. Evidence generated by this study suggested high rates of ET-induced toxicity for both, pre- and postmenopausal women. The prevalence of most side effects observed in this “real-life” study (i.e. a sample within routine after-care) significantly exceeded those reported by the original registration trials [16–18]. Joint pain, hot flashes, loss of interest in sex and lack of energy were the most prevalent symptoms reported by patients. In order to gain more insight into the long-term sequelae of ET, the authors conducted a follow-up study to the research project PRO-BETh.

The main aim of this follow-up study was the determination of patient-reported ET-associated toxicity and QOL outcomes in BCS 5–10 years after the initial assessment.

## Patients and methods

### PRO-BETh study description

The original PRO-BETh study [14, 15] was designed as a cross-sectional observation study targeting on the assessment of prevalence and severity of ET-induced side effects from a subjective patient perspective. For this purpose, BC patients undergoing up-front ET with either AIs or tamoxifen (with or without Zoladex) at the time of assessment completed a comprehensive PRO-battery on QOL including physical side effects and psychosocial burden. Reported symptom prevalence rates were compared to data derived from pivotal phase IV trials (ATAC 2005, BIG1-98 [16]. Overall, PROs resulted in significantly higher prevalence rates as compared to physician ratings for most symptoms published in pivotal clinical trials. The authors concluded that ET toxicity seems to be underestimated in clinical routine care. Please find further study details in the respective publications [11, 12].

### Sample

All BC patients who had participated in the original study were eligible and approached for participation in the follow-up assessment. Contact data were taken from the medical records of the Department of Gynecology and Obstetrics at the Medical University of Innsbruck. Inclusion criteria for this study were defined as followed:

- Participation in the initial PRO-BETh study
  Breast cancer survivor having undergone endocrine treatment - defined as patient who had completed the primary treatment (maintenance treatment can be ongoing) by the EORTC Cancer Survivorship Task Force [19]
- No overt cognitive impairment
- Written informed consent
- Fluency in German

### Procedure

Following the recruitment procedure of the original project, the data assessment was conducted at the outpatient unit of the Department of Gynecology and Obstetrics at the Medical University of Innsbruck.

Breast cancer survivors (BCS) were approached for study participation either at one of their routine follow-up appointments (in Austria, BC patients have lifelong routine check-ups at the primary care center) or via mail after an introductory telephone call explaining the study purpose. Patients completed written informed consent. In case of consenting to study participation, BCS completed the same PRO assessment used in the original study (see below). Patients returned the questionnaires pseudo-anonymized (ID indicated by a number) in an envelope either via mail or personally at the outpatient unit (paper–pencil assessment). Clinical data for participants were derived from the medical records.

### PRO instruments

The original questionnaire battery included the Functional assessment of cancer therapy-breast (FACT-B) and Functional assessment of cancer therapy-endocrine subscale (Fact-ES). The Functional Assessment of Cancer Therapy-Breast and Endocrine Subscale (FACT-B + -ES) consists of 36 items assessing QOL in BC patients. The questionnaire uses a five-point Likert scale and relates to the FIM framework for the past seven days. The answer format ranges from 0 (not at all) to 4 (very much). The maximum scoring for general well-being ranges from 0 to 108, for emotional well-being from 0 to 24 and for physical and functional well-being from 0 to 28. High values indicate a good QOL. The FACT-B is supplemented by the endocrine subscale (FACT-ES), which measures symptoms and side effects related to ET for breast cancer such as hot flashes, joint pain and loss of libido [3]. The FACT-ES comprises 19 items. Further details have been published elsewhere [14, 15].

## Statistical analysis

Sample characteristics are described using absolute and relative percentages, means and standard deviations.

Primary analysis: In order to investigate long-term ET toxicity, we analyzed the FACT-B + -ES on single item level following the analysis of the original study. i.e. we compared FACT-B + -ES data of each patient from the first assessment to her data at the follow-up assessment. We present the prevalence of patient-reported physical and psychological symptoms related to ET (derived from the FACT-B + -ES) as percentages and 95% confidence intervals for baseline and follow-up time points. Symptom frequency was calculated by summarizing percentages of patients selecting the categories '*somewhat*', *'quite a bit'* and *'very much'* on single item level of the FACT-B + -ES. Confidence intervals were calculated using the modified Wald method [20]. The Sign Test was used to compare symptom frequencies between the two assessment-time points. We further aimed at the clarification of the impact of age on symptoms. For this purpose, age was considered a relevant covariate already at the first assessment with a continuous effect on the outcome. We hence were interested in the impact of age on symptom change over time rather than assessing its effect at the follow-up assessment only. For this purpose, we calculated the difference between the first- and follow-up assessment for the FACT-B + -ES items and compared age groups (< 50, 50–59, 60–69 and > 70 years) for this difference using the Kruskal–Wallis Test.

Secondary analysis: For the investigation of overall long-term QOL outcome (i.e. FACT-G and -ES sum score), we used a mixed linear model. In this analysis, the dependent variables were log-transformed to obtain normal distribution. Time point (first assessment, follow-up) was included as a fixed effect and post-hoc, we conducted pairwise comparisons between time points and tamoxifen- vs. aromatase inhibitor treatment (with Bonferroni-correction for multiplicity). To assess the association of age with change over time we included the two-way interaction age-by-time point in the model. To account for correlations between repeated measurements, we used a first order autoregressive covariance matrix. P-values below 0.05 were considered statistically significant. All analyses were done with SPSS 22.0.

We obtained ethical approval for this follow-up project from the Ethics Committee of the Medical University of Innsbruck (Innsbruck, 22.04.2017/Ah).

## Results

### Sample

**Fig. 1:**
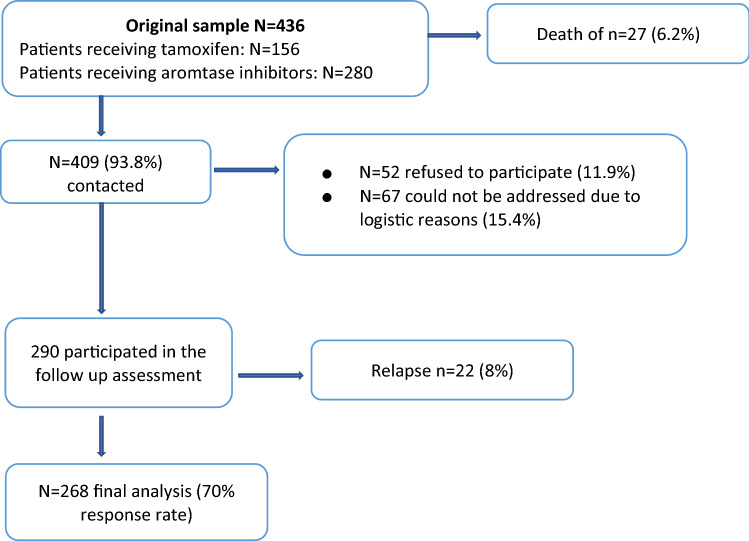
Flow diagram of the inclusion/exclusion of the study participants and response rate

From the 436 patients on endocrine treatment originally surveyed in 2009–11, 27 patients (6.2%) were deceased, this corresponds to an OS of 93%. A total of 290 breast cancer long-term survivors participated in the follow-up study. The remaining 119 patients did either not agree to fill out questionnaires because of personal reasons (11.9%) or could not be contacted due to logistic reasons (15.4%). Hence, a response rate of 70% could be achieved. Among the patients in the final analysis, a total of 8% reported a relapse (3.4% in the AI group and 4.5% in the tamoxifen group). We excluded those patients from the further analysis to provide group homogeneity. Hence, we report data of a final sample of 268 BCS. Please find details in the flow chart below (Fig. 1).

Patients participated after a median follow-up period of 8 years (range 6–9 years; mean = 8.02). At the time of the follow-up assessment, patients were aged 65 years on average and 90% were postmenopausal. Patients who had received tamoxifen were significantly younger than patients with AI therapy (*p* < 0.001) as tamoxifen has been the first-line ET for premenopausal patients at the time the original study was performed (and AIs for postmenopausal patients). Details on clinical data are presented in Table 1.

**Table 1:** Clinical patient data (*n* = 268) at follow-up

		Patients with AI treatment *N* = 159	Patients with tamoxifen treatment *N* = 109	All BCS *N* = 268
		Frequency (%)	Frequency (%)	Frequency (%)
Age at the follow-up	Mean (SD)	72.8 (SD 7.2) years^#^	54.5 (SD 5.3) years^#^	65.3 (SD 11) years
	Range	56–94	36–68	(36–94 years)
Time since diagnosis	Median (SD)	10.4 (SD 2) years	9.2 (SD 1.3) years	10 (SD 1.9) years
	Mean	10.9 years	9.4 years	10.3 (SD 1.9) years
	Range	7–17 years	7–13 years	7–17.6 years
Time between assessments	Median (SD)	8.2 (SD 0.5) years	7.9 (0.6) years	8 (SD 0.6) years
	Mean	8.2 years	7.8 years	8.0 years
	Range	6.9–9.0 years	6.4–9.10 years	6.4–9.1 years
Time between ET termination and follow-up	Median (SD)	4.9 (SD 2) years	3.8 (SD 1.8) years	4.5 (SD 2) years
	Mean	4.9 years	3.9 years	4.4 (SD 2) years
	Range	ongoing -9 years	ongoing—8 years	ongoing—9 years
Menopausal state	Premenopausal	–	27 (25%)	27 (10%)
	postmenopausal	159 (100%)	81 (75%)	240 (90%)
Overall duration of adjuvant endocrine therapy (months)	Mean (SD)	5.8 (SD 1.9) years	5.3 (SD 1.7) years	5.5 (1.8) years
	Range	2.7–15 years	1.7–13 years	1.7–15 years
ET intake (treatment duration groups)	< 5 years	10 (6.4%)	16 (14.8%)	26 (10%)
	Regular 5 years Extended	110 (70.1%)	76 (70.4%)	186 (70%)
		37 (23.5%)	16 (14.8%)	53 (20%)
ET ongoing at follow-up	yes	7 (4.4%)	8 (7.3%)	15 (5.6%)
BMI	Mean	25.6	25.6	25.7
	Range	16.9–46.9	16.9–46.9	18.2–40.8

^#^Significant difference between patients treated with tamoxifen vs. AIs regarding age (*p* < 0.001)

### Changes of ET-related toxicity

We observed a significant improvement of the overall endocrine symptomatology in the long-term (FACT-ES baseline mean = 59 vs. follow-up mean = 62, *p* < 0.001); this significant improvement was found for both, patients who had received tamoxifen (FACT-ES baseline mean = 58.4 vs. follow-up mean = 61.2) as well as those with previous AI treatment (FACT-ES baseline mean = 59.7 vs. follow-up mean = 62.7).

In detail, vasomotor symptoms including hot flashes and cold/night sweats decreased significantly with time in both groups. In contrast, gynecologic symptoms did not change over time except for vaginal discharge, which decreased significantly; loss of interest in sex even increased in long-term in percentages. Interesting to note, of the overall sample 9.3% did not complete both questions on sexuality and 20.7% answered only one of both questions (i.e. pain with intercourse and loss of interest in sex) at the second assessment.

No significant difference was observed for gastrointestinal symptomatology (*p* > 0.05 for all gastrointestinal symptoms); a total of 38.4% and 30.4% of patients reported weight problems in the tamoxifen and AI group, respectively, at follow-up. Finally, the typically ET-related symptoms joint pain, lack of energy and mood swings were highly prevalent at the follow-up assessment time point. Details are presented in Table 2, 3, 4.

**Table 2:** Prevalence physical and psychological symptoms in BCS (entire sample)

	All BCS *N* = 268		
	1st assessment	Follow-up	Difference in %
Median time since diagnosis	1.9 (SD 1.7) years	10.0 (SD 1.9) years	
	% (CI95%)	% (CI95%)	
**Vasomotor symptoms**			
Hot flashes	68.7% (62–74)	39.5% (33–46)*	**− 29.2**
Cold sweats	30.0% (24–36)	16.3% (12–21)*	**− 13.7**
Night sweats	47.0% (40–53)	24.3% (19–30)*	**− 22.7**
Sleeping difficulties	14.9% (11–20)	9.5% (6–14)^#^	**− 5.4**
**Gynecologic symptoms**			
Vaginal discharge	12.4% (9–17)	4.6% (2–8)*	**− 7.8**
Bleeding or spotting	1.6% (0.5–4)	1.4% (0.3–4)	− 0.2
Vaginal itching/irritation	9.8% (6–14)	10.3% (7–14)	+ 0.5
Vaginal dryness	30.2% (25–36)	33.0% (27–39)	+ 2.8
Breast sensitivity/tenderness	24.4% (19–30)	19.1% (14–24)	− 5.3
Pain or discomfort with intercourse	16.5% (12–22)	20.5% (15–27)	+ 4
Lost interest in sex	40.4% (34–46)	48.7% (42–55)*	** + 8.3**
**Gastrointestinal symptoms**			
Weight gain	36.8% (31–43)	33.9% (28–40)	− 2.9
Emesis	1.2% (0–3)	0.9% (0–2)	− 0.3
Diarrhoea	5.2% (3–9)	5.8% (3–10)	+ 0.6
Feeling bloated	14.7% (11–19)	9.5% (6–14)	− 5.2
Nausea	4.5% (2–8)	3.6% (2–7)	− 0.9
**Pain**			
Headaches	16.4% (12–21)	14.7% (10–20)	− 1,7
Joint pain	45.5% (40–51)	44.2% (38–50)	− 1.3
**Psychological symptoms**			
Feeling lightheaded (dizziness)	13.9% (10–19)	13.9% (10–19)	–
Mood swings	36.8% (31–43)	30.2% (24–36)*	**− 6.6**
Being irritable	30.6% (25–36)	27.5% (22–33)	− 3.1
Lack of energy	36.4% (30–42)	33.8% (28–40)	− 2.6

*Significant difference on a *p* < 0.01 (based on the Sign Test)

^#^Significant difference on a *p* < 0.05 (based on the Sign Test)

**Table 3:** Prevalence physical and psychological symptoms in BCS having received ET with tamoxifen

	Patients with TAM treatment *N* = 159		
	1st assessment	Follow-up	Difference in %
Median time since diagnosis	1.2 (SD 1.0) years	1.2 (SD 1.0) years	
	% (Cl95%)	% (Cl95%)	
Vasomotor symptoms			
Hot flashes	83.8% (75–89)	50.0% (40–59)*	**− 33.8**
Cold sweats	39.6% (30–49)	21.6% (14–30)*	**− 18.0**
Night sweats	60.6% (51–69)	29.9% (21–39)*	**− 30.7**
Sleeping difficulties	14.2% (8–22)	5.9% (2–12)*	**− 8.3**
Gynecologic symptoms			
Vaginal discharge	25.0% (17–34)	5.2% (2–12)*	− 19.8
Bleeding or spotting	3.8% (1–9)	2.1% (0–7)	− 1.7
Vaginal itching/ irritation	14.3% (8–22)	13.3% (7–21)	− 1
Vaginal dryness	21.9% (15–30)	**35.1% (26–45)#**	** + 13.2**
Breast sensitivity/ tenderness	21% (14–29)	19.4% (13–28)	− 0.6
Pain or discomfort with intercourse	14.4% (8–22)	21.5% (14–31)	+ 7.1
Lost interest in sex	21.4% (14–30)	**34.7% (26–44)**^**#**^	** + 13.3**
Gastrointestinal symptoms			
Weight gain	37.5% (29–47)	38.4% (29–48)	+ 0.9%
Emesis	1.0% (0–5)	0.0% (0)	− 1
Diarrhoea	2.9% (0.6–8)	3.0% (0.6–8)	− 0.1
Feeling bloated	14.3% (8–22)	10.2% (5–18)	− 4.1
Nausea	3.9% (1–10)	2.1% (0–7)	− 1.8
Pain			
Headaches	13.3% (8–21)	12.1% (7–20)	− 1.2
Joint pain	30.5% (22–40)	35.1% (26–45)	+ 4.6
Psychological symptoms			
Feeling lightheaded (dizziness)	8.6% (4–15)	6.1% (3–19)	− 2.5
Mood swings	38.2% (30–48)	34.0% (25–44)	− 4.2
Being irritable	34.6% (26–44)	25.8% (18–35)	− 8.8
Lack of energy	32.7% (24–42)	27.5% (20–37)	− 5.2

*Significant difference on a *p* < 0.01 (based on the Sign Test)

^#^Significant difference on a *p* < 0.05 (based on the Sign Test)

**Table 4:** Prevalence physical and psychological symptoms in BCS having received ET with aromatase inhibitors

	Patients with AI treatment *N* = 109		
	Baseline	Follow-up	Difference in %
Median time since diagnosis	2.2 (SD 1.9) years	10.4 (SD 2) years	
	% (CI95%)	% (CI95%)	
Vasomotor symptoms			
Hot flashes	57.8% (49–65)	31.2% (23–39)*	**− 26.6**
Cold sweats	23.2.% (17–30)	12.1% (7–19)*	**− 11.1**
Night sweats	37.4% (30–45)	19.8% (13–27)*	**− 17.4**
Sleeping difficulties	15.4% (10–22)	12.4% (7–19)	− 3%
Gynecologic symptoms			
Vaginal discharge	3.4% (1–8)	4.1% (1–9)	+ 0.7
Bleeding or spotting	0.0% (0)	0.8% (0–5)	+ 0.8
Vaginal itching/ irritation	7.5% (4–13)	7.1% (3–13)	− 0.4
Vaginal dryness	36.1% (28–44)	31.5% (24–40)	− 4.6
Breast sensitivity/ tenderness	26.9% (20–34)	**18.9% (12–27)#**	**− 8**
Pain or discomfort with intercourse	18.3% (12–26)	19.6% (13–29)	+ 1.3
Lost interest in sex	55.3% (46–63)	61.5% (52–70)	+ 6.2
Gastrointestinal symptoms			
Weight gain	36.3% (29–44)	30.4% (23–39)	− 5.9
Emesis	1.4% (0–5)	1.6% (0–6)	+ 0.2
Diarrhoea	6.9% (3–12)	8.0% (4–14)	+ 1.1
Feeling bloated	15.1% (10–21)	9.0% (5–15)	− 6.1
Nausea	4.9% (2–10)	4.6% (2–9)	− 0.3
Pain			
Headaches	18.6% (13–25)	16.7% (11–24)	− 1.9
Joint pain	56% (48–63)	51.2% (42–60)	− 4.8
Psychological symptoms			
Feeling lightheaded (dizziness)	17.7% (12–25)	20.0% (14–28)	+ 2.3
Mood swings	35.8% (28–43)	27.2% (20–35)	− 8.6
Being irritable	27.7% (21–35)	28.8% (21–37)	+ 1.1
Lack of energy	38.9% (31–47)	38.5% (30–47)	− 0.4

*Significant difference on a *p* < 0.01 (based on the Sign Test)

^#^Significant difference on a *p* < 0.05 (based on the Sign Test)

Regarding the effect of age—independent from the treatment received—on symptom change over time, we found no difference for most symptoms across age groups (results not shown) with the exception of vaginal discharge (*p* < 0.001), headaches (*p* = 0.023) and mood swings (*p* < 0.001) (details in Table 5).

**Table 5:** Mean symptom difference from baseline to follow-up across age groups (significant results *p* < 0.05)

Symptoms age at follow-up	vaginal discharge mean difference (SD)	Headaches mean difference (SD)	mood swings mean difference (SD)
< 50 years	− 1 (1.13)	− 0.3 (0.9)	− 0.9 (1.6)
50–59 years	− 0.4 (0.9)	0.12 (0.9)	− 0.014 (1.6)
60–69 years	− 0.23 (0.7)	− 0.35 (0.9)	0.54 (1.7)
> = 70 years	0.03 (0.5)	− 0.04 (0.9)	1 (1.5)

### QOL outcome

Overall, QOL according to the FACT-global score was significantly higher in long-term BCS compared to QOL in patients on ET treatment (mean baseline = 88.3 vs. mean follow-up = 90.9, p = 0.011). This was true for patients who had received tamoxifen (mean baseline = 89.3 vs. mean follow-up = 92.8) as well as those with previous AI treatment (mean baseline = 87.5 vs. mean follow-up = 89.5). However, in terms of clinical relevance the improvement seems to be minor [21].

BCS reported significantly higher levels of physical well-being (FACT-physical well-being baseline = 23.9 mean vs. follow-up mean = 24.8, *p* < 0.01) and functional well-being (FACT-functional well-being baseline mean = 21.7 vs. follow-up mean = 22.7, *p* = 0.013) than patients on ET treatment. For functional well-being, we observed a trend towards a higher increase in patients who had received tamoxifen, i.e. in the younger patient group (interaction effect *p* = 0.079) compared to patients in the AI-group.

No changes were observed for emotional well-being (FACT-emotional well-being baseline = 19.7 mean vs. follow-up mean = 20, *p* > 0.05) and social well-being (FACT-emotional well-being baseline mean = 22.5 vs. follow-up mean = 22.5, *p* > 0.05). This was true for both ET groups.

For all QOL scales, age had no significant impact on symptom change over time (results not shown).

## Discussion

While previous evidence suggests that ET-associated toxicity is high and distinctly impairs patient QOL during intake [14, 22] we lack evidence on the patients’ experience of these symptoms in the long run, in particular after ET termination. In this paper, we aimed at shedding light to the physical and psychosocial long-term outcome after ET in BCS from a patient perspective.

Overall, BCS experienced a decrease of the overall ET-related symptomatology in the long-term (as indicated by the overall score of the FACT-ES subscale). In particular, the vasomotor symptomatology—a major side effect of ET—seems to decrease over time significantly. In addition, patients reported a small but significant increase of the overall physical and functional well-being score as well as their general QOL over time. This observation is consistent with the results of Schmitt et al. demonstrating improvement of physical- and role-functioning in-between 5 years after the end of cancer treatment and even exceeding levels of an aged-matched healthy population [23, 24]. Others suggest levels of overall QOL in BCS to be comparable to those of a population without a previous cancer disease [7, 8]. We might hence conclude that there is some sort of stabilization of an overall symptomatology and QOL in BCS.

Consequently, we observed a lack of recovery when it comes to specific ET-related symptoms. Several symptoms seem to persist at high levels: Joint pain, loss of interest in sex or weight gain as well as lack of energy have been indicated as the most prevalent long-term follow-up problems for BCS in our study. Our results complement existing evidence illustrating specific cancer treatment-related symptoms to challenge patients in long-term. For instance, Haidinger [9] and others [25] observed high levels of joint pain in BCS after ET termination. Van Leuuwen (2018) identified joint pain among the chronic symptoms highly relevant and burdensome for cancer survivors when asking patients to quote QOL topics relevant for their cancer survivorship [19]. Evidence for the persistence of fatigue and lack of energy as among the most prevalent long-term sequels of a cancer disease is robust [7]. Weight gain is a well-known problem related to ET [26]. Particularly, patients receiving tamoxifen (i.e. younger patients) continue to struggle with their weight over years [26]. In the study presented herein, more than one third of both, AI-patients and TAM-patients, reported persistent problems with weight gain. Potentially resulting in obesity, weight gain is not only a problem for the subjective overall well-being, body image or feeling of attractiveness but mediates disease control and clinical outcome [27].

Moreover, this study again proves the impact of ET on sexual health in BCS. We observed not only a lack of recovery of interest in sexuality in long-term but even a tendency towards symptom deterioration in the “younger” (originally premenopausal) patient group. The same was true for vaginal dryness in premenopausal patients. This is in line with two recent meta-analyses highlighting a high prevalence of female sexual dysfunction in BCS [28, 29]. The authors reported recently that about 70% of BCS complain clinically relevant sexual dysfunction [30]. In addition, 10% of the participants had not answered the two questions about sexuality and 20% only answered one of the two questions. This observation supports the notion that the topic of sexuality is still a taboo in clinical care patients are reluctant to talk about [31–33]. Sexuality is a complex issue influenced by numerous bio-psycho-social factors and underlies natural changes over the life span: For instance, age or menopausal status are well-known to affect interest in sexuality or libido [34, 35]. In this study, we were not able to clearly isolate an independent effect of factors potentially contributing to the patients’ sexual outcome as we lack a baseline assessment of QOL before the start of ET. However, in view of more than 50% of BCS on- and off- treatment indicating sexual impairments, sexuality should be considered as a major, persistent care demand relevant for BC (survivorship) care.

With regard to the psychological domain, BCS reported no change over time. Established evidence supports the notion that psychological issues continue to be high far into the survivorship stage while patients recover physically [9]. Mood swings – though decreasing over time – can be a continuing problem at least for about a third of the younger patients as observed in this study. Other studies [7, 9] also describe comparable results with regard to emotional well-being. In particular, young BC patients need to meet the challenges of a cancer diagnosis in the middle of their work life, educational stage or during the phases of family planning; life plans need to be postponed or can finally not be achieved, thereby, requiring (psychological) adaptation to new requirements and are psychologically challenging. (Irreversible) hormonal changes can—as induced by ET—might aggravate the emotional challenges; the latter was not investigated in this study but will be the topic of further studies.

In conclusion, a distinct proportion of BCS experiences a chronification of specific symptoms after treatment completion. These health impairments can significantly interfere with a management of daily life independently from others thereby having a profound impact on patient QOL. The persistence of some ET-related physical and emotional symptoms should not be underestimated—this is true also beyond the actual intake of ET.

## Limitations

A limiting factor for the interpretation of study results is the lack of knowledge of QOL outcome in the non-participant group. Though we have a very satisfactory response rate of 70%, a potential selection bias towards a worse OR better QOL cannot be excluded. Furthermore, a direct comparison of data with an age-matched population without a cancer disease would have enriched the interpretability of data on QOL outcome. Data from such a reference sample could help identify other factors modulating QOL over a span of almost a decade beside the cancer diagnosis and related treatments e.g. natural menopause and age are well-known to have an effect on interest in sex or mood swings independently from cancer. The investigation of the independent effect of age on QOL outcome was further limited in the presented analysis due to the following: The type of ET prescribed originally had been based on patients’ menopausal state (i.e. tamoxifen for premenopausal patients and aromatase inhibitors for postmenopausal patients), so that age is an immanent factor related to the type of treatment (i.e. a high inter-correlation of the covariates age and type of treatment). An independent effect of age is therefore difficult to obtain. Finally, the sample heterogeneity in terms of treatment duration and time since ET termination to the follow-up assessment limits the interpretation of results. Clearly, a longitudinal design with a baseline assessment before the start of ET and a more homogenous sample at the first and second assessment would have contributed to a more accurate picture of the true extent of long-term toxicity caused by adjuvant ET—this limitation from the original study persists to the follow-up assessment. However, presented results clearly indicate that BCS experience unfavorable long-term effects that need to be better understood and should be subject to further research.

## Clinical implication

Our results are of importance for clinical survivorship care: Women after ET seem to recover well overall when it comes to QOL issues. However, they still suffer from particular health impairments presenting a high potential for QOL limitations. Most persistent problems seem to be sexual health issues, psychological demands and joint pain. Survivorship care efforts should focus on these problems. This includes the provision of more information on long-term sequel of breast cancer and ET in patient education, a systematic assessment of the respective symptoms at after-care visits, and the integration of targeted, supportive treatment individually tailored to the BCSs’ demands in long-term care plans. This might also include the adjustment of ET treatment application towards individual demands. For instance, the SOLE study [36] proved an intermittent administration of Letrozole as a safe and advantageous option in terms of QOL. The option for a treatment interruption might help patients to stabilize their OOL and ultimately better adhere to the treatment regime. Beside the increase of survival, the prevention of long-term QOL problems should be an ultimate goal for BC survivorship care.

## Appendix

See Table

**Table 6:** Physical and psychological symptoms in BCS at the first and the follow-up assessment

	All BCS *N* = 268			
Symptoms	Symptom persistence^a^ (%)	Symptom present at first but not at follow-up assessment (%)	Symptom not present a first but at follow-up assessment (%)	Total symptom prevalence (%)
Vasomotor symptoms				
Hot flashes	34.4	36.3	5.1	39.5
Cold sweats	8.6	23	7.7	16.3
Night sweats	17.2	29.7	7.1	24.3
Sleeping difficulties	5.8	9.8	4	9.5
Gynecologic symptoms				
Vaginal discharge	2.9	11.5	1.7	4.6
Bleeding or spotting	1.4	1.4	0	1.4
Vaginal itching/irritation	4.8	6.5	5.5	10.3
Vaginal dryness	18.4	13.7	15.1	33.0
Breast sensitivity/tenderness	16.1	8.5	10.4	19.1
Pain or discomfort with intercourse	8	6.8	12.3	20.5
Lost interest in sex	28.5	10.2	20.4	48.7
Gastrointestinal symptoms				
Weight gain	17.8	18.6	17.3	33.9
Emesis	0.9	1.4	0	0.9
Diarrhoea	0.5	4.2	5.1	5.8
Feeling bloated	3.8	11.3	6.1	9.5
Nausea	3.6	4.4	0	3.6
Pain				
Headaches	7.4	10.2	7	14.7
Joint pain	29.8	14.2	14.2	44.2
Psychological symptoms				
Feeling lightheaded (dizziness)	5.6	9.3	7.9	13.9
Mood swings	19.9	17.5	9.5	30.2
Being irritable	15	15	11.7	27.5
Lack of energy	18.8	17	14.8	33.8

^a^Group of patients with the respective symptom at both assessment time points.

^b^Group of patients with the respective symptom at the first assessment only (not reported at the follow-up assessment).

^c^Group of patients with the respective symptom at the follow-up assessment only (not reported at the first assessment).

6.
